# A comparison of thoracic or lumbar patient-controlled epidural analgesia methods after thoracic surgery

**DOI:** 10.1186/1477-7819-12-96

**Published:** 2014-05-04

**Authors:** Gonul Sagiroglu, Burhan Meydan, Elif Copuroglu, Ayse Baysal, Yener Yoruk, Yekta Altemur Karamustafaoglu, Serhat Huseyin

**Affiliations:** 1 Department of Anesthesiology, Faculty of Medicine Houses, Trakya University, D- Bloc, No: 8, Edirne, Turkey; 2 Department of Anesthesiology, Sureyyapasa Chest Disease and Thoracic Surgery Hospital, Istanbul, Turkey; 3 Department of Anesthesiology, Kartal Kosuyolu Yuksek Ihtisas Training and Research Hospital, Istanbul, Turkey; 4 Department of Thoracic Surgery, Faculty of Medicine, Trakya University, Edirne, Turkey; 5 Department of Cardiovascular Surgery, Faculty of Medicine, Trakya University, Edirne, Turkey

**Keywords:** Thoracic epidural analgesia, Lumbar epidural analgesia, Cardiac enzymes, Visual analog scale

## Abstract

**Background:**

We aimed to compare patient-controlled thoracic or lumbar epidural analgesia methods after thoracotomy operations.

**Methods:**

One hundred and twenty patients were prospectively randomized to receive either thoracic epidural analgesia (TEA group) or lumbar epidural analgesia (LEA group). In both groups, epidural catheters were administered. Hemodynamic measurements, visual analog scale scores at rest (VAS-R) and after coughing (VAS-C), analgesic consumption, and side effects were compared at 0, 2, 4, 8, 16, and 24 hours postoperatively.

**Results:**

The VAS-R and VAS-C values were lower in the TEA group in comparison to the LEA group at 2, 4, 8, and 16 hours after surgery (for VAS-R, *P* = 0.001, *P* = 0.01, *P* = 0.008, and *P* = 0.029, respectively; and for VAS-C, *P* = 0.035, *P* = 0.023, *P* = 0.002, and *P* = 0.037, respectively). Total 24-hour analgesic consumption was different between groups (175 +/- 20 mL versus 185 +/- 31 mL; *P* = 0.034). The comparison of postoperative complications revealed that the incidence of hypotension (21/57, 36.8% versus 8/63, 12.7%; *P* = 0.002), bradycardia (9/57, 15.8% versus 2/63, 3.2%; *P* = 0.017), atelectasis (1/57, 1.8% versus 7/63, 11.1%; *P* = 0.04), and the need for intensive care unit (ICU) treatment (0/57, 0% versus 5/63, 7.9%; *P* = 0.03) were lower in the TEA group in comparison to the LEA group.

**Conclusions:**

TEA has beneficial hemostatic effects in comparison to LEA after thoracotomies along with more satisfactory pain relief profile.

## Background

The traumatic process of a thoracotomy operation involves cutting of the muscles, separation of the ribs along with the use of retractors during the procedure, and acute pain is expected to occur after surgery due to damage to the intercostal nerves. For postoperative pain management, either lumbar or thoracic epidural analgesia is required to diminish the incidence rate of possible pulmonary and cardiac complications [1,2]. However, the use of nerve blocks do not provide sufficient analgesia, and there is a need for additional analgesics to prevent break through pain [3,4].

The lumbar epidural analgesia (LEA) method is an alternative method for thoracic epidural analgesia (TEA) for the treatment of post-thoracotomy pain [1,5,6]. The use of the TEA method was reported as a superior method in comparison to LEA due to better pain control and diminished incidence of postoperative complications [2-4,7]. TEA reduces cardiac and splanchnic sympathetic activity and thereby influences perioperative function of vital organ systems. A recent meta-analysis suggested that TEA decreased postoperative cardiac morbidity and mortality. TEA appears to ameliorate gut injury in major surgery as long as the systemic hemodynamic effects of TEA are adequately controlled. The risk of harm by TEA is even lower, and other methods used to control perioperative pain and stress response also carry specific risks [8].

Our goal was to compare the hemodynamic and analgesic effects of patient-controlled thoracic or lumbar epidural analgesia methods in a prospective randomized study design after thoracotomy operations.

## Methods

**Figure 1:**
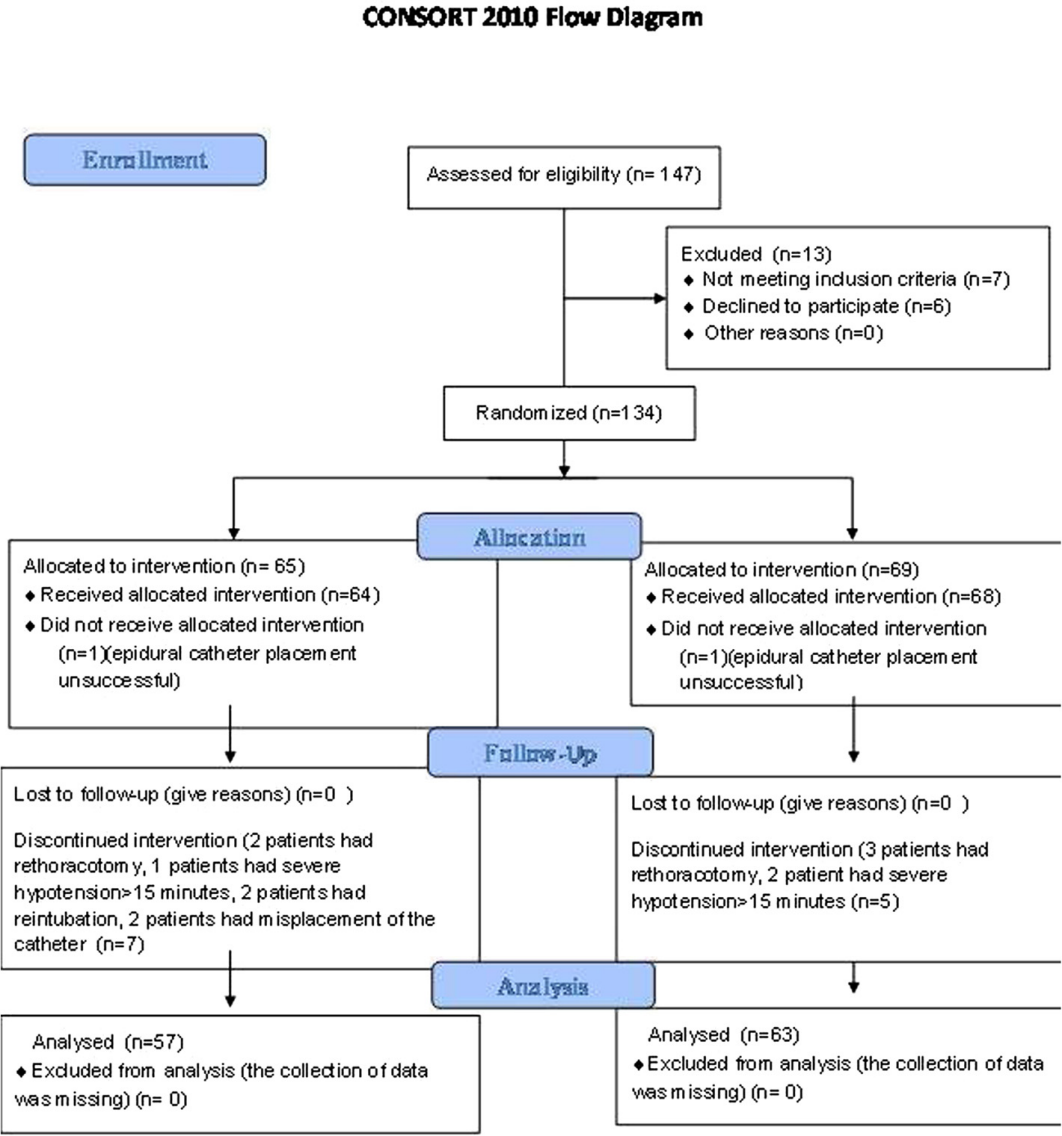
Consort 2010 flow diagram.

Institutional Review Board of Sureyyapasa Chest Disease and Thoracic Surgery Hospital, clearance and written informed patient consent were obtained. One hundred and twenty adult patients with a diagnosis of lung cancer underwent a posterolateral thoracotomy procedure by one surgeon. Most incisions were performed with posterolateral thoracotomy because of an advanced stage of lung cancer and a huge mass. Muscle-sparing thoracotomy was applied when possible. Patients in an age range of 46 to 86 years and of American Society of Anesthesiologists (ASA) physical status I, II or III were included in a prospective, randomized, double-blind controlled study. The allocation of patients into groups was performed according to a random numbers list. Patients were randomized to either the thoracic epidural (TEA, n = 57) group or lumbar epidural (LEA, n = 63) group for a 24-hour postoperative period after surgery. From 147 patients, 13 were excluded as 7 of them did not meet the inclusion criteria and 6 of them declined to participate. A total of 134 patients were randomized into two groups. Two patients did not receive allocated intervention, and intervention was discontinued in 12 patients. The Consort diagram is presented in Figure 1. Exclusion criteria include: 1) ASA physical status > III; 2) known drug allergies; 3) prior lumbar spine surgery; 4) pregnancy; 5) abnormal coagulation tests such as platelet count < 80.000, prothrombin time > 1.5 min, or partial thromboplastin time > 45 s; 6) history of comorbidities such as clinical and laboratory findings of hepatic or renal disease, valvular heart disfunction, or chronic obstructive lung disease; or 7) neurological impairment causing inability to understand consent form or pain measurement.

Demographic data, age, height, weight, body mass index (BMI), cigarette smoking, pulmonary function parameters, histological type of cancer, surgical procedures and duration of operation were recorded. Preoperative pulmonary functions measured included forced expiratory volume in one second (FEV_1)_ and forced vital capacity (FVC). FEV_1_ (% predicted) and FVC (% predicted) values were determined. In addition, preoperatively, intraoperatively, and at 2, 12 and 24 hours after surgery, arterial blood gas values including pH, PaO_2_ (arterial partial pressure of oxygen) and PCO_2_ (arterial partial pressure of carbon dioxide) were collected. Also, the Charlson comorbidity index was used in this study, and it is classified into four groups to define and grade any existence of comorbidities. There are nineteen conditions that are related to increased risk for mortality and a weighted score of 1 to 6 was provided depending on the relative mortality risk.

All patients were premedicated with oral diazepam 0.1 mg/kg on the night before surgery. A five-lead electrocardiography along with an ST segment analysis provided continuous heart rate (HR) monitoring. An ST segment increase or decrease of ≥ 0.2 mV in men or ≥ 0.15 mV in women was monitored to determine signs of myocardial ischemia.

The epidural catheters were applied at the operating table prior to the induction of anesthesia. An analgesic solution that contains 0.125% bupivacaine with sufentanyl (Sufentanil, Janssen Pharmaceutical, Belgium) (0.6 μg/mL) was prepared by an anesthesia technician. All epidural punctures were inserted with an 18 gauge Touhy needle (B-Braun Medical, Abbott, Turkey) through T_4-6_ or L_2-3_ interspaces while patients were in a sitting position. The epidural space was identified by using the loss of resistance to saline technique, which is required to demonstrate a negative aspiration of cerebrospinal fluid and blood. Afterwards, a test dose of 20 mg of 2% lidocaine (Jetmonal 2%, Adeka Pharmaceutical, Turkey) with epinephrine (1: 200,000) was administered. The epidural catheter (Portex Epidural Minipack; Smiths Medical ASD Inc, Keene, NH, USA) was advanced in a cephalad direction and 5 mL was administered to both groups from the epidural catheter before general anesthesia was administered from the solution prepared before catheterization. A basal infusion at a dose of 2 mL/h was started intraoperatively after the anesthesia induction and just before surgery. At the end of the surgery, a patient-controlled epidural analgesia device (PCEA, Abbott Pain Management Provider, North Chicago, IL, USA) was connected to the epidural catheter. The infusion rate of PCEA was 0.1 mL/kg/h, with 2 mL on demand, and a lock-out interval of 30 min in the 24 hours postoperative period.

General anesthesia was induced with 1 μg/kg of fentanyl (Janssen fentanyl, Janssen Pharmaceutical, Belgium) and 5 mg/kg of thiopental (Pental, IE Ulugay Pharmaceutical, Turkey) intravenously. Tracheal intubation was facilitated with 0.15 mg/kg vecuronium (Blok-L, Mustafa Nevzat Pharmaceutical, Turkey), and repeated intraoperatively if necessary. Anesthesia was maintained with desflurane (Suprane, Baxter, Pharmaceutical, Puerto Rico, USA) in 70% oxygen and 30% air. The oxygen concentration was adjusted according to arterial blood gas values of partial arterial oxygen concentration value (PaO_2_). A left-sided double-lumen endobronchial tube was inserted. Correct tube position was confirmed with the help of a stethoscope and confirmed by flexible fiberoptic bronchoscopy in both the supine and lateral decubitus positions. For intraoperative monitoring, when mean arterial blood pressure (MAP) or HR was 25% higher than the baseline value, it was treated with 0.3 μg/kg of fentanyl. On completion of surgery, the patients were reversed with intravenous neostigmine and atropine at a dose of 0.04 mg/kg and 0.02 mg/kg, respectively.

During the first 24-hour period in the intensive care unit (ICU), MAP, HR and SpO_2_ values were recorded at 0, 2, 4, 8, 16, and 24 hours. First hemodynamic measurements in the ICU were recorded as 0 hour values. At any time point, an episode of hypotension was defined as a fall in MAP more than 25% of baseline value for a period of less than 15 minutes. Severe hypotension was a fall in MAP more than 25% of baseline value for a period more than 30 minutes. Both of these conditions were treated with Ringer lactate crystalloid fluid infusions. If infusion of crystalloid solution did not raise the blood pressure, a 10 mg ephedrine (Ephedrine, Osel Pharmaceutical, Turkey) bolus was administered and repeated if necessary. Bradycardia was defined as heart rate below 50 beats/min and was treated with bolus doses of 0.4 mg atropine sulfate (Atropin sulfate, Biofarma Pharmaceutical, Turkey).

At the end of surgery, patients were weaned from mechanical ventilation. Reintubation was necessary in some patients in the ICU. Criteria for weaning from mechanical ventilation include the following: 1) PaO_2_ ≥ 60 mmHg, 2) FiO_2_ ≤ 0.40, 3) peak end expiratory pressure (PEEP) ≤ 5 cm H_2_O, 4) respiratory rate (RR) < 30, 5) minute ventilation of < 12 L to maintain partial arterial carbondioxide tension (PaCO_2_) between 35 and 45 mmHg, 6) appropriate level of consciousness, 7) intact cough and gag reflex, 8) vital capacity > 10 mL/kg, and 9) minimum inspiratory pressure < -30 cm H_2_O.

Postoperatively, the incidence of nausea or vomiting was noted. Nausea greater than 2/10 (measured by the visual analog score (VAS)) or vomiting were treated with 10 mg intravenous metoclopramide (Metpamid, Mefar Pharmaceutical, Turkey). Pruritus was treated by intravenous difenhydramine (Benison, Osel Pharmaceutical, Turkey) 10 mg. The doses were repeated if necessary.

We administered intravenous 1,000 mg paracetamol (Perfalgan, Bristol-Myers Squibb Pharmaceutical, France) every 8 h to all patients during the ICU treatment. At a VAS score > 3, breakthrough pain relief was provided through bolus doses of epidural analgesic agent at a dose of 0.1 mL/kg in addition to PCA. Additional pain relief was provided with intravenous morphine at a dose of 2 mg (Morphine HCL, Galen Pharmaceutical, Turkey) in both groups for a VAS score > 3 at rest despite four consecutive PCA boluses.

After extubation, patients were assessed by a physician in ICU to evaluate their pain at a scale from 0 (no pain) to 10 (disabling pain) by the use of VAS. Postoperative pain scores at rest (VAS-R) and after a strong cough (VAS-C) were evaluated. Sedation scores were judged by the observer (0 = awake; 1 = mild sedation; 2 = moderate sedation, easily rousable; 3 = heavily sedated, difficult to rouse; and 4 = over-sedated, unarousable). In each case, pain and sedation scores were evaluated at 0, 2, 4, 8, 16, and 24 h by a blinded resident to the study protocol on call in the ICU.

Excessive sedation was defined as a score higher than 3 or 4 and either respiratory depression (defined as a ventilatory frequency below 8 breaths/min) or hypercarbia (PaCO_2_ > 50 mmHg) treated with 100% oxygen supplementation via a face mask. Patients were discharged from the ICU when the following criteria were met: SpO_2_ ≥ 90% at FiO_2_ ≤ 0.5 by face mask; hemodynamic parameters including MAP, HR and RR within normal adult limits; chest tube drainage < 50 mL/h; urine output > 0.5 mL/kg/h; and no intravenous inotropic or vasopressor therapy.

### Primary and secondary study endpoints

The TEA and LEA groups were compared in the first 24-hour ICU stay for 1) hemodynamic data including MAP, HR and arterial oxygen saturation (SaO_2_) values that were recorded at 0, 2, 4, 8, 16, and 24 hours; 2) visual analog scale (VAS) score at rest (VAS-R) and after coughing (VAS-C); 3) sedation scores; 4) the total amount of analgesic (PCEA) and morphine consumption; 5) the determination of postoperative complications (respiratory failure, atelectasis, reintubation, postoperative bleeding, bronchopleural fistula, rethoracotomy, in-hospital stay and 30-day mortality); and 6) the record of analgesic- and morphine-related side effects such as nausea and vomiting, pruritus, sedation or other adverse events.

### Statistical analysis

The Statistics Package for Social Sciences (SPSS 16.0; SPSS Inc., Chicago, USA) was used for all calculations. During sample size evaluation, the aim was to detect a clinically relevant reduction in the VAS score by 30 mm. To be able to detect a difference of 20 cm/h in the area under the curve of the VAS score after coughing with an expected standard deviation of 50 cm/h, and α and β errors of 0.05 (two-sided hypothesis) at a power of 0.8, the calculated sample size was 60 patients. To compensate for unforeseen dropouts and a possibly higher variability than expected, we planned to study 80 patients. The compliance of the data of the continuous measurements to a normal distribution was examined by the Kolmogorov-Smirnov test. Group differences for categorical variables were examined by Chi-square or Fisher’s exact test, as appropriate. Whereas in cases of normal distribution, group differences for continuous data were examined by independent samples *t-*test, for non-normally distributed data a Mann Whitney *U-*test was applied. Mann-Whitney U-tests were used to compare nonparametric variables between independent study groups and the Wilcoxon test for comparison between dependent groups. The difference in the time courses of parameters between groups was analyzed by two-way analysis of variance (ANOVA) for repeated measures. Results are expressed as mean ± standard deviation (SD) where appropriate. For all tests, *P* < 0.05 was considered significant.

## Results

The comparison of the baseline characteristics between groups showed no significant difference (Table 1). The distribution of the histologic type of cancer including squamous cell carcinoma, adenocarcinoma and undifferentiated large cell cancer were not different between the TEA group (n (%)): 33(58), 19(33.9), and 5(8.8), respectively, and the LEA group: 41(65), 16(25.4) and 6(9.5), respectively. The distribution of surgical procedures were not different between the TEA group (n (%)): 42(74), 10(18), and 5(8.8), respectively, and the LEA group, 37(59), 17(27), and 9(14.3), respectively. The surgical procedure was a right-sided thoracotomy in most of the cases: in the TEA group (n (%)), 45(78.9) versus in the LEA group, 42(66.7). Also, preoperative spirometric data including FEV_1_ (79.56 ± 13.92 versus 83.02 ± 13.75) and FVC (81.58 ± 16.86 versus 84.92 ± 15.33) values were not different between the TEA and LEA groups.

**Table 1:** Baseline clinical characteristics of the study population

	**TEA group (n = 57)**	**LEA group (n = 63)**	*P
Age (years)	55.37 ± 13.3	52.73 ± 13.33	0.281
Height (cm)	167.77 ± 7.87	166.4 ± 10.705	0.428
Weight (kg)	69.39 ± 12.44	7 4.24 ± 15.83	0.066
Body mass index (kg/m^2^)	24.6 ± 3.58	25.14 ± 4.9	0.492
**Gender (F/M)**			
Female	7 (12.3)	9 (14.3)	0.747
Male	50 (87.7)	54 (85.7)	
**ASA status**			
I	6 (10.5)	10 (15.9)	0.39
II	40 (70.2)	36 (57.1)	0.139
III	11 (19.3)	17 (27)	0.32
**Charlson comorbidity index**			
0	6 (10.5)	9 (14.3)	0.534
1- 2	38 (66.7)	40 (63.5)	0.716
> 2	13 (22.8)	14 (22.2)	0.939

*TEA*, thoracic epidural group; *LEA*, lumbar epidural group; *ASA*, American Society of Anesthesiologists physical status.

*P < 0.05, values are mean ± standard deviation, n (%), number (percent).

Comparisons of arterial blood gas values of PaO_2_ and PCO_2_, serum CK-MB and troponin values preoperatively, and postoperatively at 6 and 24 hours, did not show statistically significant differences between groups (Table 2).

**Table 2:** **The comparison of PaO**_**2**_**, PaCO**_**2**_**, serum CK-MB and troponin values preoperatively and postoperatively at 6 and 24 hour between groups**

	**Preoperative**	*P	**Postoperative 6th hour**	*P	**Postoperative 24th hour**	*P
PaO_2_ (mmHg)						
TEA	126.54 ± 33.75	0.252	146.88 ± 51.2	0.185	135.37 ± 56.65	0.149
LEA	135.86 ± 51.89		134.67 ± 49.12		151.21 ± 62.17	
PaCO_2_ (mmHg)						
TEA	41.86 ± 8.28	0.152	41.32 ± 9.39	0.131	42.11 ± 7.43	0.059
LEA	39.46 ± 9.78		43.84 ± 7.7		44.89 ± 8.47	
CK-MB (U/L)						
TEA	18.5 (13-55)	0.623	22.5 (12-46)	0.243	17.5 (16-35)	0.061
LEA	20 (12-25)		16 (11-26)		19.5 (23-35)	
Troponin (ng/mL)						
TEA	0.02 (0.01-0.6)	0.758	0.3 (0.08-0.75)	0.326	0.2 (0.05-0.60)	0.503
LEA	0.04 (0.01-0.25)		0.25 (0.09-0.55)		0.15 (0.06-0.75)	

*TEA*, thoracic epidural group; *LEA*, lumbar epidural group; *PaO*_*2*_, arterial partial pressure of oxygen; *PaCO*_*2*_, arterial partial pressure of carbon dioxide; *P < 0.05, values are mean ± standard deviation or median (minimum-maximum).

The observers were blinded to the epidural analgesia protocol. Caregivers were not blinded, but they did not participate in data collection or data interpretation.

The postoperative hemodynamic data for MAP, HR, RR, and SpO_2_ were not significantly different at any time interval between the TEA and LEA groups. Two patients (n/63, %) (2/63, 3.2%) in the LEA group had ST segment depression during and after surgery. In contrast, none of the patients in the TEA group had signs of myocardial ischemia during or after surgery. Another patient in the LEA group developed a severe depression in the ST segment (-0.2 mV) six hours after operation in the ICU. A comparison of creatinine kinase MB fraction (CK-MB) and troponin values at different time points is shown in Table 2.

A comparison of VAS-R and VAS-C scores is presented in Tables 3 and 4. Between-group comparisons revealed that VAS-R and VAS-C values were lower in the TEA group in comparison to the LEA group at 2, 4, 8, and 16 hours after surgery (for VAS-R, *P* = 0.001, *P* = 0.01, *P* = 0.008, and *P* = 0.037 and for VAS-C, *P* = 0.035, *P* = 0.023, *P* = 0.002, and *P* = 0.029; respectively). However, at 0 and 24-hour time points, the observed differences were not statistically significant.

**Table 3:** The comparison of visual analog scale (VAS) scores at rest at different time points

**Time (hours)**	**Visual analogue scala-rest**		**Grup TEA vs. Grup LEA (P)**	**The comparation intragroup of postoperative TEA (P)**	**The comparation intragroup of postoperative LEA (P)**
	**TEA group (n = 57)**	**LEA group (n = 63)**			
**Basal**	5.43 ± 1.8	5.36 ± 1.85	0.826	**Δ**	**Δ**
**2**	4.28 ± 1.59	5.33 ± 1.88	0.001^a^	< 0.0001^b^	< 0.0001^c^
**4**	3.84 ± 1.84	4.84 ± 2.3	0.01^a^	< 0.0001^b^	< 0.0001^c^
**8**	1.86 ± 1.97	2.84 ± 2.02	0.008^a^	< 0.0001^b^	< 0.0001^c^
**16**	1.72 ± 1.81	2.49 ± 2.17	0.037	< 0.0001^b^	< 0.0001^c^
**24**	1.51 ± 1.72	1.6 ± 1.77	0.768	< 0.0001^b^	< 0.0001^c^

*TEA*, thoracic epidural group; *LEA*, lumbar epidural group; Values mean ± standard deviation.

^*a*^Comparation between groups, P < 0.05.

^*b*^Comparation to basal value in Grup TEA, P < 0.05.

^*c*^Comparation to basal value in Grup LEA, P < 0.05.

**Table 4:** The comparison of visual analog scale (VAS) scores at during coughing at different time points

**Time (hours)**	**Visual Analogue Scala-Coughing**		**Grup TEA vs. Grup LEA (P)**	**The comparation intragroup of postoperative TEA (P)**	**The comparation intragroup of postoperative LEA (P)**
	**TEA group (n = 57)**	**LEA group (n = 63)**			
**Basal**	7.75 ± 1.48	7.32 ± 1.52	0.114	**Δ**	**Δ**
**2**	5.23 ± 1.96	6.02 ± 2.07	0.035^a^	< 0.0001^b^	< 0.0001^c^
**4**	4.72 ± 2.47	5.81 ± 2.7	0.023^a^	< 0.0001^b^	< 0.0001^c^
**8**	3.18 ± 0.66	4.1 ± 2.09	0.002^a^	< 0.0001^b^	< 0.0001^c^
**16**	3.54 ± 1.72	4.03 ± 1.78	0.029	< 0.0001^b^	< 0.0001^c^
**24**	3.21 ± 1.7	3.81 ± 2.01	0.082	< 0.0001^b^	< 0.0001^c^

*TEA*, thoracic epidural group; *LEA*, lumbar epidural group; Values mean ± standard deviation.

^*a*^Comparation between groups, P < 0.05.

^*b*^Comparation to basal value in Grup TEA, P < 0.05.

^*c*^Comparation to basal value in Grup LEA, P < 0.05.

The comparisons within groups for VAS-R scores revealed that the scores at 0 hours (5.43 ± 1.8) in the TEA group were higher than the VAS-R scores at 2 hours (4.28 ± 1.59; *P* < 0.0001), 4 hours (3.84 ± 1.84; *P* < 0.0001), 8 hours (1.86 ± 1.97; *P* < 0.0001), 16 hours (1.72 ± 1.81; *P* < 0.0001), and 24 hours (1.51 ± 1.72; *P* < 0.0001) for the same group. VAS-R scores at 0 hours (5.36 ± 1.85) in the LEA group were higher than VAS-R scores at 2 hours (5.33 ± 1.88; *P* = 0.038), 4 hours (4.84 ± 2.3; *P* < 0.0001), 8 hours (2.84 ± 2.02; *P* < 0.0001), 16 hours (2.49 ± 2.17; *P* < 0.0001), and 24 hours (1.6 ± 1.77; *P* < 0.0001) of the same group.

Within group comparisons revealed that the VAS-C scores at 0 hours (7.75 ± 1.48) in the TEA group were higher than the VAS-C scores at 2 hours (5.23 ± 1.96; *P* < 0.0001), 4 hours (4.72 ± 2.47; *P* < 0.0001), 8 hours (3.18 ± 0.66; *P* < 0.0001), 16 hours (3.54 ± 1.72; *P* < 0.0001), and 24 hours (3.21 ± 1.7; *P* < 0.0001) in the same group. VAS-C scores at 0 hours (7.32 ± 1.52) in the LEA group were higher than VAS-C scores at 2 hours (6.02 ± 2.07; *P* = 0.038), 4 hours (5.81 ± 2.7; *P* < 0.0001), 8 hours (4.1 ± 2.09; *P* < 0.0001), 16 hours (4.03 ± 1.78; *P* < 0.0001), and 24 hours (3.81 ± 2.01; *P* < 0.0001) of the same group.

While total 24-hour analgesic consumption was different between groups (175 ± 20 mL versus 185 ± 31 mL; *P* = 0.034), morphine consumption was similar (8.2 ± 11.3 mg versus 10.3 ± 11 mg). There was no difference in the use of ephedrine (19.29 ± 9.97 mg versus 12 ± 4.47 mg) and atropine (0.61 ± 0.22 mg versus 0.5 ± 0.00) between groups. Sedation scores were similar at 0, 2, 4, 8, 16 and 24 hours postoperatively. Two patients (3.2%) in the LEA group versus none in the TEA group had a sedation score ≥ 3 for 24 hours.

The incidence of hypotension and bradycardia and the need for an ICU stay > 24 hours are presented in Table 5. Severe hypotension was observed in only one patient in each group of patients. The incidence of nausea and vomiting of the TEA group (4/57, 7%) was lower than the LEA group (7/63, 11.1%). Three patients with vomiting received metoclopramide treatment. There was no incidence of any other complications in both of the groups. Urinary retention could not be assessed, since patients routinely had Foley catheters inserted at the time of surgery.

**Table 5:** The comparison of analgesic related adverse events and postoperative complications between groups

	**Group TEA (n = 57)**	**Group LEA (n = 63)**	P*
Hypotension episode	8 (12.7)	21 (36.8)	0.002*
Bradycardia	2 (3.2)	9 (15.8)	0.017*
Nausea and vomiting	7 (11.1)	4 (7)	0.438
ICU stay > 24 hr	0 (0)	5 (7.9)	0.031*
Atelectasis	1 (1.8)	7 (11.1)	0.042*
Pneumonia	2 (3.2)	3 (5.3)	0.567
Reintubation	2 (3.2)	0	0.175
Bronchopleural fistula	1 (1.6)	2 (3.5)	0.501
Rethoracotomy	2 (3.2)	3 (5.3)	0.567
Permanent neurological dysfunction	0	0	NS

*P < 0.05, (n,%), Hypotension episode: fall in systolic blood pressure more than 25*%* of baseline value for a period of less than 15 minutes. *ICU*, intensive care unit; *LEA*, lumbar epidural group; *TEA*, thoracic epidural group; *NS*, not significant.

The duration of operation time of the TEA group was similar to that for the LEA group (222.34 ± 74.1 min versus 204.4 ± 64.1 min). The patients in the TEA group were discharged home in a comparable time period to the LEA group (8.74 ± 5.62 days versus 9.17 ± 6.83 days).

## Discussion

In the early postoperative period, the use of a PCEA through either a lumber or thoracic epidural catheter has been shown to decrease the risk of myocardial ischemia, improve lung ventilation and decrease incidences of atelectasis and pneumonia [2,3,7]. In our study, a major finding is that the incidence of hypotension and bradycardia were significantly lower in the TEA group in comparison to the LEA group. However, we have shown no difference in the evaluation of serum CK-MB, troponin values or ST segment analysis in a period of 24 hours postoperatively. In a recent meta-analysis including 2,758 patients and nine studies, it was demonstrated that TEA did not reduce perioperative myocardial ischemia or mortality in patients undergoing noncardiac operations [9]. However, there are limited data on perioperative outcome comparisons of patients undergoing thoracotomy procedure who had either a lumbar or thoracic epidural for postoperative pain management. A recent study investigated fifty-five patients with blunt chest wall trauma who were randomized to receive an epidural morphine injection once daily for 24 hours through a lumbar or thoracic catheter [10]. In this study, in a comparison of two groups, no differences were found regarding incidences of cardiac or pulmonary complications or the occurrence of epidural morphine-related side effects. Forty patients undergoing coronary artery bypass graft (CABG) with normal left ventricular ejection fraction were randomized to receive epidural buprenorphine for pain relief following extubation. For breakthrough pain, intramuscular ketorolac tromethamine at a dose of 30 mg was administered. In this study group, both groups had comparable cardiac and pulmonary functions postoperatively in addition to comparable side effects and complications [11]. In another small randomized study, the efficacy of thoracic epidural sufentanil 50 micrograms was compared with lumbar epidural sufentanil 50 micrograms in an equally distributed group of 30 patients. Although the number of patients who had hypotensive episodes was higher in the TEA group, the study showed that both techniques are comparable regarding pain relief, complications and side effects [12].

Previous studies investigated the effects of TEA on left ventricular function, and in these studies, major concerns of TEA were 1) a decrease in preload related to venodilation, 2) impairment in cardiac contractility secondary to sympathectomy at T_4_ dermatome level, and 3) a decrease in heart rate secondary to either sympathectomy or increased vagal tone [13,14]. There is also sympathetic activity during surgery and postoperative pain that is associated with increased myocardial oxygen consumption [15]. Other studies demonstrated that lumbar epidural analgesia, when used during surgery, may cause a major problem. This is mainly reflex arterial dilatation and bradycardia via the Bezold-Jarisch reflex, which induces events such as a reduced myocardial blood flow distal from coronary artery stenosis, a possible increase of oxygen demand by sympathetic activation in nonblocked thoracic segments, and an impairment of myocardial wall motion [16]. Recently it has been suggested that, as there is a need to restrict splanchnic sympathetic block, maintain venous return, and lessen hypotension, lumbar epidural anesthesia should be avoided in patients undergoing abdominal or thoracic procedures [17,18].

In our study, the incidences of hypotension and bradycardia were significantly higher in the LEA group in comparison to the TEA group. In a recent study, a significantly higher incidence of hypotension with lumbar PCEA (7.7%) was observed compared with thoracic PCEA (4.1%) (*P* = 0.001) [15]. We also demonstrated that perioperative myocardial ischemia was not significantly different between patients receiving either TEA or LEA, which was shown by serum CK-MB, troponin values and ST segment analysis. In a comparison of TEA and LEA in abdominal and thoracic procedures, a significant reduction in myocardial infarction and an improvement of ischemia induced left ventricular global and regional wall motion abnormalities with use of TEA were demonstrated [19-21]. Also, a significantly reduced incidence of supraventricular tachycardia after both cardiac surgery and pulmonary resection with the use of TEA was reported. However, we were not able to demonstrate this because we used TEA for postoperative analgesia only and not during the operation [21-23]. A previous study of the use of TEA for postoperative analgesia after thoracotomy is not available for a comparison of the cardioprotective effects of LEA.

The other most prominent action of TEA is on pulmonary functions. TEA has been associated with earlier mobilization, reduced opioid consumption, and improved cough, and thus, it has beneficial effects on lung functions causing diminished incidences of atelectasis or pneumonia [15,24]. In another meta-analysis including only CABG, patients reported faster extubation and less pulmonary complications with thoracic epidural blockade [23]. In our study, we showed that TEA causes reduced incidence of atelectasis in comparison to LEA. No permanent neurological sequelae were reported in a total of 4,185 patients with use of TEA; however, temporary neurological sequelae such as peripheral nerve injury or dural tap-related headache are much more frequent [24].

The comparison of TEA and LEA for pain relief after thoracotomies in the early postoperative period revealed that both techniques provide efficient analgesia; however, TEA has superior pain relief profile. These findings are comparable with previous studies and meta-analyses [2-4,11,15,19,21].

## Conclusions

Although better cardioprotective effects on serum cardiac enzymes and ST segment analysis have not been shown, TEA has beneficial hemodynamic effects in comparison to LEA after thoracotomies along with a more satisfactory pain relief profile in the 24-hour postoperative period.

## Abbreviations

BMI: body mass index; CABG: coronary artery bypass graft; CK-MB: creatinine kinase MB fraction; FEV1: forced expiratory volume in one second; FVC: forced vital capacity; HR: heart rate; ICU: intensive care unit; LEA: lumbar epidural analgesia; MAP: mean arterial blood pressure; PaO_2_: arterial partial pressure of oxygen; PCEA: patient-controlled epidural analgesia device; PCO_2_: arterial partial pressure of carbon dioxide; RR: respiratory rate; TEA: thoracic epidural analgesia; VAS: visual analog score; VAS-C: visual analog score after coughing; VAS-R: visual analog score at rest.

## Competing interests

The authors declare that they have no competing interests.

## Authors’ contributions

All authors read and approved the final manuscript.
